# Examination of experienced coaches and physical education teachers' teaching methods and their perceptions regarding these methods—2023

**DOI:** 10.3389/fspor.2024.1383361

**Published:** 2024-06-03

**Authors:** Şengül Demiral, Meltem Nazıroğlu

**Affiliations:** ^1^ Department of Coaching Education, Kırkpınar Faculty of Sport Science, Trakya University, Edirne, Türkiye; ^2^ Institute of Health Sciences, Trakya University, Edirne, Türkiye

**Keywords:** coaching, teaching, methods, perception, experiences

## Abstract

**Introduction:**

It is widely acknowledged that coaches and physical education teachers play an important role in supporting holistic development in children and ensuring optimal performance in the training processes carried out to acquire fundamental movements and sport-specific basic skills. However, there is a need for further information on how both groups utilize and value different teaching methods during the training. The present study aims to examine the perceptions of coaches and physical education teachers regarding the use and value of teaching methods.

**Methods:**

The “Coaches’ Instructional Methods Utilization Scale” for coaches and the “Physical Education Teachers’ Perception of Teaching Methods Scale” for physical education teachers were administered to 114 coaches and 115 physical education teachers voluntarily participating from three randomly selected provinces of Türkiye. The Cronbach Alpha values ranged between .89 and .93 for the “Coaches’ Instructional Methods Utilization Scale” and between .90 and .96 for the “Physical Education Teachers’ Perception of Teaching Methods Scale”. Descriptive statistics were used in research, *t*-tests in binary comparisons, One-Way ANOVA in multiple comparisons, and Tukey's test in determining the source of differences.

**Results:**

Similarities were observed in the most and least used methods by coaches and physical education teachers, as well as in their perceptions of the highest and lowest values for these methods. Additionally, coaches and physical education teachers exhibited similarities in their perceptions of value in terms of entertainment, learning, and motivation. It was observed that female physical education teachers had lower value perceptions among the levels of use of teaching methods and value perceptions according to gender. Comparing the usage levels and value perceptions of teaching methods by professional experience, significant differences were found in Exercise (B), Learner-Designed Individual Program (I), and Learner-initiated (J) methods for coaches, whereas no statistically significant difference was observed in value perceptions. Moreover, considering the physical education teachers, significant differences were found in Command (A), Self-Check (D), Guided Discovery (F), Problem-Solving: Single Solution (G) methods, and in value perceptions for the Exercise (B), Guided Discovery (F), Problem-Solving: Single Solution (G), Problem-Solving: Crating Different Paths (H), and Learner-initiated (J) values.

## 1 Introduction

Physical education teachers can determine the teaching methods they prefer to use while developing their personal teaching theories (1). Simultaneously, as coaching began to be perceived as a “pedagogical” profession (2) and coaches started to approach athlete development with an “educational perspective” (3), they may also identify the teaching methods they prefer to use. Due to a limited number of comparative studies examining teaching methods in the literature of sports sciences within physical education and sports education, this study aims to investigate the teaching methods used by experienced coaches and physical education teachers and their perceptions regarding these methods.

The most prominent study addressing teaching approaches and methods in physical education is the study carried out by Mosston and Ashworth's (4) “Teaching Styles.” This study categorizes teaching approaches into two main categories: “Command” and “Discovery,” encompassing a total of 11 teaching methods (5 under “Command” and 6 under “Discovery”). These teaching methods are widely accepted as “Teaching Methods” in the literature of physical education. Mosston's teaching spectrum includes 11 methods reflecting behavioral, cognitive, and constructivist approaches (4). These methods include (A) Command, (B) Practice, (C) Reciprocal, (D) Self-Check, (E) Inclusion, (F) Guided Discovery, (G) Problem-Solving: Single Solution, (H) Problem-Solving: Creating Different Paths Production, (I) Learner-Designed Individual Program, (J) Learner-initiated, and (K) Self-Teaching. In Mosston's classification, Methods A, B, C, D, and E are mainly teacher-oriented ones, whereas Methods F, G, H, I, J, and K are those, in which the learner is more active (5). While behavioral approach-based teaching methods can be effective in mastering fundamental skills, they may fall short in acquiring higher-level skills such as complex problem-solving, critical thinking, and deduction (6). In contrast, cognitive-based teaching methods (F, G, H) focus more on learners' cognitive activities such as attention and information coding, considering learners' thoughts, beliefs, and values in the learning process (7). The learner is expected to make the information meaningful for himself/herself and relate it to his/her existing knowledge by organizing the newly acquired information. When compared to behavioral approaches, cognitive-based teaching methods can provide more in-depth and advanced learning environments since they consider learners' cognitive processes and social characteristics (8, 9).

Lev Vygotsky (9) focuses on the social learning theory in his work. He emphasizes how instructional methods are associated with students' social interactions and learning environments. Therefore, we can conclude that coaches’ instructional methods are shaped based on the type of learning or learning environment. Moreover, Vygotsky highlights how coaches' instructional methods shape students' learning environments and emphasizes the outcomes of this interaction. Additionally, we can conclude that coaches' instructional methods are determined based on students' social interactions and environmental factors (8, 9).

Teachers' use of instructional models, approaches, and methods, and their effectiveness on learning are highly debated and investigated in the field of education. Furthermore, it is thought that learning objectives that are changing and becoming more complex over time cannot be achieved by solely using theory-based models, approaches, and methods (10–15). Reviewing the literature on coaching, it is emphasized that the physical, psychosocial, and motor development of children and young people should be supported to achieve lifelong participation in sports and healthy high-level talent development (16), that coaching started to be perceived as a “pedagogical” profession (2), and that coaches should approach athlete development with an “educational perspective” (3, 17–22). It is expected that athletes, as participants and competitors, should ensure cognitive, emotional, and psychomotor development. Particularly, through sports participation, children and young people are expected to develop competence in sports-related skills (such as technical, tactical, and physical skills), self-esteem, self-confidence, positive relationships with coaches and others, and character traits such as respect, honesty, empathy, and a sense of responsibility (10). The realization of these sports achievements is closely related to the level of coaches’ ability to structure sports education methods and environments by considering the developmental levels of athletes and the requirements of the sports environment. Examining the current athlete development models, such as Long-Term Athlete Development (LTAD) (23) and the Developmental Model of Sport Participation (DMSP) (16), it is emphasized that sports education should offer developmental age-appropriate physical, psychological, social, and cognitive developmental opportunities with an inclusive, long-term, and athlete-centered approach for superior performance and healthy development in sports.

The Long-Term Athlete Development Model (LTAD) is a model that emphasizes the importance of considering individual differences in biological maturity rather than chronological age when programming for youth and starting the training process in early childhood. The model aims to provide a comprehensive approach to enable young athletes to engage in sports in a healthy and sustainable manner. It supports athletes’ physical, technical, tactical, and psychological development while also aiming to create a long-term athlete development plan (24–26).

The LTAD model provides different levels of programs tailored to athletes' ages and developmental stages. At the Fundamental Stage, which is typically suitable for children aged 6–9, the focus is on developing fundamental physical skills such as coordination, flexibility, and balance. At the Developmental Stage (Technical and Tactical Development), the aim is to enhance athletes' technical and tactical skills in the sport. Typically, suitable for athletes aged 10–14, this stage emphasizes further development of fundamental skills and gaining competitive game experience. At the Advanced Stage (Performance and Competition), the focus shifts to helping athletes become high-performance competitive athletes. Targeting athletes aged 15 and above, this stage aims to maximize athletes' physical, technical, tactical, and psychological skills to compete at the highest level (23).

Regardless of whether the focus is on participation or talent development, the LTAD model aims to monitor and support athletes' long-term success. This model adopts a balanced approach to prevent potential issues such as overtraining or injury risk. Additionally, the LTAD model helps athletes create a customized training and development plan tailored to their ages, developmental stages, and goals. The Long-Term Athlete Development Model has been adopted and implemented by numerous sports organizations and federations worldwide. The success of this model stems from its consideration of individual needs and goals, ensuring that athletes engage in sports more healthily and successfully (27).

The Developmental Model of Sport Participation (DMSP) covers 3 developmental stages. It considers 6–12 years as experimentation years, 13–15 years as specialization years, and 16 years and above as career years. With this model, it recommends trying various sports during childhood (16) and allocating more time to structured educational games in the 6–12 age range. Structured play refers to early exploratory physical activities that are inherently motivated and primarily aimed at maximizing pleasure and fun (16). The International Society of Sport Psychology's position on the 6–15 age range (28) emphasizes that it has positive effects for long sport careers and long-term sport participation, positively affects youth development, forms the basis of intrinsic motivation, and provides motor and cognitive experience (29).

For this purpose, it is thought that coaches' professional competencies in the field and their pedagogical approach in sports play an important role. Moreover, teaching methods are shaped based on the type of learning content and environment, and who makes decisions on the learning. Based on these factors, teaching methods are generally established on behavioral, social-cognitive, and constructivist learning theories. Behavioral theories provide teacher-centered knowledge accumulation, whereas social-cognitive and constructivist theories predominantly present learner-oriented knowledge. Coaches' teaching methods are limited to their knowledge and their awareness of the results these methods may yield in effective learning for athletes (3).

Therefore, it is important for coaches and physical education teachers to have a high awareness of teaching methods estimating the changing needs and in-depth knowledge about teaching methods shedding light on relevant learning theories that serve learning. Hence, Mosston's Spectrum of Teaching Styles provides a conceptual framework for coaches and physical education teachers to develop awareness about teaching method options and determine conceptual areas of need related to these options (4, 30). Through Mosston's Spectrum of Teaching Styles, coaches and physical education teachers can adopt the most effective teaching methods that cater to the needs of the sports environment from a broader perspective and implement the most suitable teaching method for the sports environment. These methods can be applied in various areas of sports education (4, 30, 31). However, teaching models, approaches, and methods developed within the “General Education” field generally apply to the “Physical Education and Sports” field as well, but they have been reshaped within this field's structure based on kinesthetic development and teaching through physical exercises.

Metzler (32) defined 8 different instructional models in the field of physical education, namely “Direct,” “Individualized,” “Cooperative,” “Sports Education,” “Peer,” “Cognitive,” “Tactical Game,” and “Personal and Social Responsibility.” According to the constructivist approach, knowledge is not independent of the mind and is produced based on personal experiences. Learning is integrated with the learning content and environment (33). Therefore, in the constructivist approach, learners are expected to actively experience realistic situations related to learning in an environment directly associated with learning, solve various problems, and ultimately produce knowledge specific to their environment.

According to Jonassen (34), there are three levels of knowledge acquisition: entry-level, advanced, and upper-level. Instructional methods based on objective approaches (Behaviorist approach) are more effective in responding to entry-level learning, whereas it is necessary to resort to constructivist instructional methods (I, J, and K), where the learner is actively at the center of the learning process in real environments, for higher-level knowledge acquisition, which involves solving complex and unstructured problems. The use of athlete-centered instructional methods reflecting the constructivist approach in sports environments is very important for children and young people to develop “autonomy” in the long run. Besides its significant role in acquiring advanced skills, decision-making, and problem-solving abilities, it is well-known that coach practices promoting autonomy in athletes are highly effective in enhancing the entertainment of sports, fostering a commitment to sports, and reducing negative perceptions even when exposed to physically intense loads (35, 36). Therefore, current studies on coaching revealed that autonomy-developing sports environments increase athletes' entertainment of sports and their perception of commitment (37). Coaching practices should be able to respond to needs that arise depending on the characteristics of athletes, the requirements of the environment, and the content of the skills taught. At this point, each teaching method in Mosston's Spectrum of Teaching Styles can be designed in an autonomy-developing manner (3), with a focus on progressively developing more autonomy in learners from Method A to Method K (4).

However, it can be seen that there are studies in the literature that explain the predominant use of behaviorist-oriented teaching methods by physical education teachers and coaches (3, 38, 39). Understanding coaches' knowledge and usage levels of teaching methods from a coach's perspective is very important for optimal athlete development in all sports environments. Furthermore, it can be seen in the literature that there are studies investigating the use of teaching methods by coaches by making use of coaches' perceptions (40).

Studies examining sports education primarily focus on teachers' practices (39) and coaches' behaviors (41, 42) in physical education and coach development environments. While the studies examining coaching behaviours in the literature emphasise that teaching is the most preferred and motivating form of coaching practice by athletes, it is also pointed out that coaches may lack the knowledge and skills to apply teaching methods for different learning situations. As a result, the education provided to young athletes may fall short in meeting their specific learning needs (43). More in-depth studies on the teaching methods, tendencies, and perceptions of coaches and physical education teachers are necessary in order to improve the effectiveness of coaching and physical education practices. Considering the advancing technology and science, the literature on specific coaching methods applied by coaches and teaching methods of physical education teachers should also be thoroughly reviewed.

To summarise, understanding physical education teachers' experiences and perceptions about teaching methods can contribute to the development of effective practices in education and increase student achievement (44, 45).

These studies will be helpful in designing professional development programs for experienced coaches and physical education teachers to enhance their professional knowledge for improving advanced learning in children's chronological and biologically defined developmental areas in sports sciences.

Taking into account the above-mentioned justifications, this study aims to examine the usage levels of teaching methods and value perceptions regarding these methods among experienced coaches and physical education teachers. The following research questions were formulated:

(1) What are the usage levels of teaching methods and the value perceptions regarding these methods among experienced coaches and physical education teachers?
(2) Are there differences in the value perceptions of experienced coaches and physical education teachers in the dimensions of Entertainment, Learning, and Motivation?
(3) Are there gender differences in the usage levels of teaching methods and the value perceptions among experienced coaches and physical education teachers?
(4) Are there differences in the usage levels of teaching methods and value perceptions among experienced coaches and physical education teachers based on professional experience?

## 2 Materials and methods

### 2.1 Participants

Using the convenience sampling method, the present study involved randomly selected 114 coaches (58 males, 56 females) and 115 physical education teachers (77 males, 38 females) actively engaged in coaching and physical education teaching in the provinces of Edirne, Tekirdağ, and Kırklareli in the year 2023, having a minimum of 5 years of experience, and volunteering to participate. Because Ericson (46) and Balyi et al. (23) stated in his studies that it takes at least 10 years to specialize in a field, we took 5 years of experience as a reference since the scales we used in our study were developed on experienced coaches and physical education teachers.

### 2.2 Data collection instruments

The Concurrent Validity and Reliability of Coaches' Use of Teaching Methods Scale: Coach Version (CUTEMS—Coach) and the “Physical Education Teachers’ Perceptions of Teaching Styles” scales, deemed appropriate by the Trakya University Social and Humanities Research Ethics Committee in its meeting on 18 October 2023 (Decree Nr. 09/12) were utilized.

“Concurrent Validity and Reliability of Coaches’ Use of Teaching Methods Scale: Coach Version (CUTEMS—Coach) (47)” and “Physical Education Teachers’ Perceptions of Teaching Styles” (48) were used. The Cronbach's alpha internal consistency coefficient for the factors of the Concurrent Validity and Reliability of Coaches' Use of Teaching Methods Scale: Coach Version (CUTEMS—Coach) is 0.72, 0.78, and 0.76 for each factor, respectively. The validity and reliability of the scale (40, 47) were improved based on athlete perception. The scale consists of 11 teaching methods, each assessed through four questions on a 5-point Likert scale (Never, Rarely, Occasionally, Frequently, Always). The first question measures the extent to which coaches use the instructional method, while the others address the value perception associated with each teaching method.

The “Physical Education Teachers’ Perceptions of Teaching Styles” scale, designed specifically for teachers, includes 11 instructional styles and 11 factors (39), whereas the scale used for coaches combines 11 styles into three factors (47). Both scales utilize a 5-point Likert scale for evaluation. The value perception levels are examined by calculating the average of the total score from the three items related to value perception (min.3, max.15). The usage of teaching methods and value perceptions of styles are scored on a scale from 1–5 for the lowest and highest values that can be obtained, respectively (47).

### 2.3 Data collection procedures

Before data collection, permission was obtained from the Research Ethics Committee of Trakya University (No:09/12.). Participants included active coaches and physical education teachers with a minimum of 5 years of experience in Edirne, Tekirdağ, and Kırklareli in 2023. The researcher collected data by visiting sports clubs and school environments. Findings obtained from the sample are only generalizable to this specific group. The limitations of studies conducted using the survey method, both in terms of data collection and the findings related to instructional methods and their value perceptions, should be considered. Surveys were administered in person by visiting work environments, utilizing both digital (tablet) and paper-based methods.

### 2.4 Data analysis

The data were matched in terms of participant and survey numbers. Before the analysis of data, checks for missing and outlier values were conducted, and skewness and kurtosis values were examined. Based on these considerations, the decision to apply parametric tests was made. Descriptive statistics, *t*-tests for binary comparisons, One-Way ANOVA for multiple comparisons, and the Tukey test to determine the source of differences were used for data analysis. The Cronbach's alpha values ranged from .89–.93 for the “Coaches’ Instructional Methods Utilization Scale” and from .90–.96 for the “Physical Education Teachers’ Instructional Styles Value Perceptions” scale in this study.

## 3 Results

Rq1: What are the usage levels of teaching methods and the value perceptions regarding these methods among experienced coaches and physical education teachers?

According to the first research question, descriptive statistical results of the average usage levels and value perceptions for each method indicate that coaches predominantly employ the “command,” “exercise,” and “participation” methods, while using the “student initiation” and “self-teaching” methods to a lesser extent. The most highly valued methods are found to be “exercise,” “command,” and “reciprocal,” whereas the least valued methods are identified as “student initiation” and “self-teaching.”

According to physical education teachers, they predominantly use the “command” and “exercise” methods, with the least utilization of the “student initiation” and “self-teaching” methods. The most highly valued methods are “exercise” and “command,” while the least valued methods are “student initiation” and “self-teaching” (Table 1).

Rq2: Are there differences in the value perceptions of Entertainment, Learning, and Motivation dimensions among Experienced Coaches and Physical Education Teachers?

**Table 1:** Average usage levels and value perceptions of teaching methods and methods used by experienced coaches and physical education teachers.

Methods	Coaches		Physical education teachers	
	Usage*	Value perceptions**	Usage*	Value perceptions**
	Mean (SD)	Mean (SD)	Mean (SD)	Mean (SD)
Command (A)	3.86 (1.14)	12.10 (2.52)	4.32 (0.78)	12.81 (2.30)
Exercise (B)	3.57 (1.16)	11.96 (2.71)	3.84 (1.15)	11.91 (3.21)
Reciprocal (C)	3.26 (1.08)	11.22 (2.50)	3.04 (1.11)	10.43 (2.82)
Self-Check (D)	3.13 (1.09)	10.19 (2.76)	2.74 (1.27)	9.36 (3.71)
Participation (E)	3.44 (1.03)	10.88 (2.66)	3.13 (1.35)	10.33 (3.61)
Guided discovery (F)	3.39 (1.14)	10.60 (2.77)	2.92 (1.31)	9.87 (3.52)
Problem-Solving: Single Truth (G)	3.15 (1.10)	10.87 (2.78)	2.82 (1.25)	9.52 (3.30)
Problem-Solving: Creating Different Paths (H)	3.31 (1.15)	10.78 (2.53)	2.85 (1.21)	9.42 (3.19)
Learner-Designed Individual Program (I)	2.99 (1.10)	9.85 (2.72)	2.78 (1.27)	9.62 (3.57)
Learner-initiated (J)	2.62 (1.17)	8.94 (3.20)	2.48 (1.37)	8.38 (3.96)
Self-teaching (K)	2.28 (1.14)	8.21 (3.43)	1.72 (1.16)	6.06 (3.75)

* min. 1, max. 5.

** min. 3, max. 15.

Considering the 2nd research question, separately examining the items related to entertainment, learning, and motivation within the value perceptions of coaches, it was observed that the “Command (A)” and “Exercise (B)” methods are the most highly valued ones in terms of entertainment, learning, and motivation, whereas the least valued methods in terms of entertainment, learning, and motivation were determined to be the “Learner-initiated (J)” and “Self-teaching (K)” methods. Examining the items related to entertainment, learning, and motivation within the value perceptions of physical education teachers, it was observed that the “command” and “exercise” methods were the most highly valued ones in terms of entertainment, learning, and motivation, while the least valued methods in terms of entertainment, learning, and motivation were the “learner-initiated” and “self-teaching” methods (Table 2).

Rq3: Is there a difference in the usage levels and value perceptions of teaching methods based on gender among experienced coaches and physical education teachers?

**Table 2:** Ranking of average value perceptions in the entertainment, learning, and motivation dimensions among experienced coaches and physical education teachers from high to Low.

	Coaches			Physical education teachers		
	Entertainment	Learning	Motivation	Entertainment	Learning	Motivation
	Mean (SD)	Mean (SD)	Mean (SD)	Mean (SD)	Mean (SD)	Mean (SD)
A	3.81 (1.06)	4.06 (0.93)	4.26 (0.92)	4.13 (0.93)	4.28 (0.85)	4.39 (0.78)
B	3.77 (1.16)	3.92 (0.95)	4.22 (0.89)	3.94 (1.20)	3.99 (0.99)	3.97 (1.18)
E	3.71 (0.98)	3.71 (0.92)	3.92 (0.87)	3.41 (0.97)	3.48 (1.17)	3.60 (1.04)
C	3.57 (0.97)	3.65 (1.00)	3.80 (1.04)	3.36 (1.29)	3.40 (0.96)	3.47 (1.26)
H	3.45 (0.91)	3.52 (0.91)	3.79 (0.93)	3.20 (1.23)	3.24 (1.21)	3.42 (1.20)
G	3.42 (1.00)	3.51 (0.97)	3.68 (1.01)	3.13 (1.12)	3.29 (1.19)	3.24 (1.15)
F	3.40 (1.01)	3.50 (1.00)	3.67 (1.02)	3.10 (1.25)	3.18 (1.12)	3.22 (1.30)
D	3.24 (1.00)	3.37 (0.98)	3.57 (1.03)	3.09 (1.18)	3.18 (1.08)	3.19 (1.29)
I	3.18 (0.95)	3.27 (0.93)	3.39 (1.06)	3.06 (1.23)	3.11 (1.27)	3.11 (1.09)
J	2.82 (1.05)	3.04 (1.13)	3.07 (1.22)	2.70 (1.35)	2.84 (1.35)	2.83 (1.39)
K	2.73 (1.19)	2.69 (1.12)	2.78 (1.25)	2.01 (1.29)	2.02 (1.30)	2.03 (1.28)

In each dimension, the minimum score is 1 and the highest score is 5.

Considering the 3rd research question, examining the *t*-test results of independent groups conducted to compare the preferred teaching methods of experienced coaches, there was no significant difference by gender (*p* > 0.05) (Table 3).

**Table 3:** Comparison of the usage levels and value perceptions of teaching methods based on gender among experienced coaches.

Method	Coaches						
	Sex	Usage			Value perceptions		
		Mean	SD	*t*	Mean	SD	*t*
A	Female *_N_*_ = 56_	3.67	1.349	−1.738	12.16	2.647	.229
	Male *_N_*_ = 58_	4.05	.886		12.05	2.423	
B	Female	3.51	1.250	−.548	11.98	2.597	.066
	Male	3.63	1.087		11.94	2.843	
C	Female	3.23	1.190	−.300	11.32	2.750	.390
	Male	3.29	.973		11.13	2.259	
D	Female	3.08	1.164	−.407	9.92	3.068	−1.005
	Male	3.17	1.011		10.44	2.429	
E	Female	3.46	1.094	.171	11.07	2.715	.728
	Male	3.43	.975		10.70	2.629	
F	Female	3.26	1.103	−1.168	10.26	2.901	−1.278
	Male	3.51	1.173		10.93	2.634	
G	Female	3.01	1.271	−1.321	10.58	2.952	−1.084
	Male	3.29	.917		11.15	2.614	
H	Female	3.35	1.227	.391	11.12	2.676	1.432
	Male	3.27	.969		10.44	2.363	
I	Female	2.94	1.181	−.425	10.01	2.540	.641
	Male	3.03	1.025		9.68	2.909	
J	Female	2.50	.972	−1.108	8.55	2.904	−1.295
	Male	2.74	1.331		9.32	3.445	
K	Female	2.25	1.066	−.362	8.01	3.142	−.614
	Male	2.32	1.219		8.41	3.713	

However, considering the independent groups *t*-test results to compare physical education teaching methods by gender, significant differences were found between men and women in the Exercise (B), Self-Check (D), Participation (E), and Individual Design (I) methods. It was observed that the Exercise and Individual Design methods were utilized more by men compared to female candidates. Gender differences were also identified in the value perceptions of methods for both men and women, specifically in the Exercise (B), Self-Check (D), Participation (E), and Individual Design (I) methods. It was determined that women had lower value perceptions for methods showing significant differences (Table 4).

Rq4: Is there a difference in the usage levels and value perceptions of teaching methods among experienced coaches and physical education teachers based on professional experience?

**Table 4:** Comparison of experienced physical education teachers’ usage levels and value perceptions of teaching methods by gender.

Method	Sex	Usage			Value perception		
		Mean	SD	*t*	Mean	SD	*t*
A	Female *_N_*_ = 38_	4.23	.819	−.809	12.81	2.216	−.005
	Male *_N_*_ = 77_	4.36	.776		12.82	2.354	
B	Female	3.47	1.288	**−2**.292*	10.89	3.790	**−2**.198*
	Male	4.02	1.050		12.41	2.783	
C	Female	2.92	1.049	−.829	10.00	2.449	−1.162
	Male	3.10	1.142		10.64	2.981	
D	Female	2.55	1.155	−1.154	8.28	3.229	**−2**.219*
	Male	2.84	1.328		9.89	3.840	
E	Female	2.84	1.461	−1.662	9.28	3.819	**−2**.204*
	Male	3.28	1.286		10.84	3.422	
F	Female	2.81	1.159	−.603	9.42	3.546	−.978
	Male	2.97	1.395		10.10	3.511	
G	Female	2.94	1.012	812	9.60	3.183	.189
	Male	2.76	1.364		9.48	3.389	
H	Female	2.94	1.038	588	9.42	2.928	−.012
	Male	2.80	1.298		9.43	3.342	
I	Female	2.36	1.148	**−2**.501*	8.05	3.578	**−3**.468*
	Male	2.98	1.292		10.40	3.337	
J	Female	2.34	1.341	−.794	7.81	3.896	−1.078
	Male	2.55	1.390		8.66	3.992	
K	Female	1.60	1.079	−.751	5.81	3.368	−.490
	Male	1.77	1.209		6.18	3.952	

*p < 0.05 (bold value).

Usage: The lowest value that can be taken is 1 and the highest value is 5.

Value Perceptions: The lowest value that can be taken is 3 and the highest value is 15.

Considering the 4th research question, the results of the One-Way ANOVA-Tukey test conducted to compare the preferred methods of coaches by their professional experience revealed significant differences in the Exercise (B), Learner-designed Individual Program (I), and Learner-initiated (J) methods (Table 5).

**Table 5:** Comparison of usage levels and value perceptions of teaching methods Among experienced coaches based on professional experience.

Method	Professional experience	Usage			Significant difference	Value Perceptions			Significant difference
		Mean	SD	*f*		Mean	SD	*f*	
A	5 years	3.70	1.337	.760	-	11.30	3.400	1.987	-
	10 years	3.69	1.262			11.57	2.398		
	11 years and longer	3.97	1.068			12.46	2.413		
B	5 years	**3**.**20**	**1**.**316**	**5**.288*	b–c	**11**.**00**	**3**.**366**	**9**.424*	b–c
	10 years	**3**.**12**	**1**.**243**			**10**.**54**	**2**.**927**		
	11 years and longer	**3**.**84**	**1**.**037**			**12**.**76**	**2**.**187**		
C	5 years	2.70	.948	1.738	-	10.20	3.190	1.044	-
	10 years	3.21	1.268			11.15	2.693		
	11 years and longer	3.36	.989			11.40	2.302		
D	5 years	2.60	.843	1.502	-	9.80	2.616	.147	-
	10 years	3.09	.947			10.12	2.534		
	11 years and longer	3.22	1.161			10,28	2.909		
E	5 years	3.10	1.370	.790	-	10.90	3.510	.387	-
	10 years	3.39	.998			10.54	2.278		
	11 years and longer	3.52	.997			11.04	2.727		
F	5 years	3.40	1.349	.423	-	10.10	3.604	.563	-
	10 years	3.24	1.061			10.30	2.391		
	11 years and longer	3.46	1.156			10.81	2.835		
G	5 years	2.90	1.286	1.770	-	9.90	3.178	.809	-
	10 years	3.45	.971			11.18	2.662		
	11 years and longer	3.05	1.132			10.87	2.797		
H	5 years	3.10	1.449	.846	-	10.20	2.973	.425	-
	10 years	3.51	1.003			11.03	2.800		
	11 years and longer	3.25	1.091			10.74	2.358		
I	5 years	**2**.**50**	**1**.**178**	**3**.331*	a–b	10.20	3.190	1.420	-
	10 years	**3**.**36**	**1**.**055**			10.45	2,538		
	11 years and longer	**2**.**88**	**1**.**076**			9.52	2.730		
J	5 years	**1**.**80**	.**918**	**3**.209*	a–b	830	2.626	.227	-
	10 years	**2**.**84**	**1**.**034**			9.06	2.633		
	11 years and longer	**2**.**63**	**1**.**221**			8.98	3.523		
K	5 years	2.70	1.159	.834	-	8.30	3.093	.316	-
	10 years	2.33	1.216			7.81	3.273		
	11 years and longer	2.21	1.107			8.39	3.579		

Groups: a: 5 years, b: 10 years, c: 11 years and longer.

*p < 0.05 (bold value).

Usage: The lowest value that can be taken is 1 and the highest value is 5.

Value Perceptions: The lowest value that can be taken is 3 and the highest value is 15.

Examining the results achieved from the One-Way ANOVA-Tukey test, conducted to compare the preferred methods of physical education teachers by their professional experience, it was determined that there were significant differences in the usage levels of the Command (A), Self-Check (D), Guided discovery (F), Problem-Solving: Single Solution (G) methods. Furthermore, significant differences are observed in value perceptions for the Exercise (B), Guided Discovery (F), Problem-Solving: Single Solution (G), Problem-Solving: Creating Different Paths (H), and Student Initiation (J) methods (Table 6).

**Table 6:** Comparison of experienced physical education teachers’ usage levels and value perceptions of teaching methods by professional experience.

Style	Professional experience	Usage			Significant difference	Value perceptions			Significant difference
		Mean	SD	*f*		Mean	SD	*f*	
A	5 years *_N_*_ = 9_	**4**.**77**	.**666**	**4**.735*	b–c	12.77	3.492	1.909	-
	10 years *_N_*_ = 41_	**4**.**51**	.**553**			13.36	1.799		
	11 years and longer *_N_*_ = 65_	**4**.**13**	.**881**			12.47	2.359		
B	5 years	4.33	1.118	2.449	-	**14**.**11**	**1**.**166**	**4**.138*	a–c
	10 years	4.04	1.047			**12**.**43**	**2**.**863**		
	11 years and longer	3.64	1.204			**11**.**27**	**3**.**443**		
C	5 years	3.44	1.130	.663	-	11.77	2.773	1.322	-
	10 years	2.97	1.060			10.09	2.643		
	11 years and longer	3.03	1.145			10.46	2.921		
D	5 years	**3**.**44**	**1**.**509**	**3**.964*	a–c	10.44	4.003	1.380	-
	10 years	**3**.**02**	**1**.**193**			9.90	3.088		
	11 years and longer	**2**.**47**	**1**.**238**			8.87	4.001		
E	5 years	4.00	1.322	2.124	-	13.00	2.061	2.815	-
	10 years	3.14	1.352			10.26	3.420		
	11 years and longer	3.01	1.340			10.00	3.787		
F	5 years	**3**.**88**	**1**.**364**	**3**.242*	a–c	**14**.**22**	**1**.**394**	**8**.602*	a–b
	10 years	**3**.**00**	**1**.**360**			**9**.**75**	**3**.**686**		
	11 years and longer	**2**.**73**	**1**.**240**			**9**.**35**	**3**.**227**		a–c
G	5 years	**3**.**87**	**1**.**125**	**3**.391*	a–c	**12**.**33**	**2**.**692**	**3**.954*	a–c
	10 years	**2**.**85**	**1**.**256**			**9**.**56**	**3**.**428**		
	11 years and longer	**2**.**67**	**1**.**226**			**9**.**10**	**3**.**157**		
H	5 years	3.77	1.301	3.084	-	**12**.**66**	**2**.**500**	**5**.476*	a–b
	10 years	2.85	1.1305			**9**.**29**	**3**.**010**		
	11 years and longer	2.72	1.218			**9,06**	**3**.**186**		a–c
I	5 years	3.55	1.589	2.222	-	11,44	4.475	1.522	-
	10 years	2.85	1.256			9,78	3.446		
	11 years and longer	2.63	1.219			9.27	3.506		
J	5 years	3.33	1.414	1.931	-	11.77	3.800	**3**.755*	a–b
	10 years	2.36	1.444			8.10	4.205		
	11 years and longer	2.44	1.299			8.09	3.656		a–c
K	5 years	2.22	1.715	1.018	-	7.33	5.099	.671	-
	10 years	1.60	1.069			5.73	3.626		
	11 years and longer	1.72	1.138			6.09	3.660		

Groups: a: 5 years, b: 10 years, c: 11 years and longer.

*p < 0.05 (bold value).

Usage: The lowest value that can be taken is 1 and the highest value is 5.

Value Perceptions: The lowest value that can be taken is 3 and the highest value is 15.

## 4 Discussion

This study aims to examine the teaching methods used by experienced coaches and physical education teachers and their value perceptions regarding these methods. Given the descriptive statistical results for each method, experienced coaches and teachers indicate that they most frequently utilize the “command,” “exercise,” and “participation” methods, but they were found to use the “learner-initiated” and “self-teaching” methods to a lesser extent. Similarities were observed in their value perceptions regarding these methods; the highest value was given to “exercise” and “command,” and the least value was given to “learner-initiated” and “self-teaching.” The results achieved here align with previous studies in the literature (49, 50). Moreover, the results support earlier relevant studies and other perspectives emphasizing the methods predominantly used by coaches during training sessions (3, 51, 52).

Experienced coaches and physical education teachers select appropriate methods based on the work environment and the student's higher-level learning needs. However, the adequacy in meeting the learning needs of students with different readiness levels and from different age groups might be questionable, and it is suggested that its effects on students' development should also be taken into account by considering their age (43, 53). Using teaching methods that align with students' cognitive, psychological, social, and emotional learning needs can be of critical importance for effective student learning (3, 54). While experienced coaches and physical education teachers place the highest value on the “command” and “exercise” methods in terms of “entertainment, learning, and motivation,” the least valued methods were determined to be “learner-initiated” and “self-teaching.” However, it is highlighted in the literature that, unlike coaches, athletes significantly perceive athlete-initiated teaching methods as more enjoyable, motivating, and instructive, emphasizing a high demand for athlete-centered teaching approaches (55, 56). Therefore, to achieve more enjoyable learning and motivation, athletes' demands should be considered. Reflecting on teaching experiences using athlete-initiated teaching methods is believed to help coaches enhance their teaching methods, ultimately improving athletes' holistic developmental sports outcomes.

However, the value given to methods in line with the results of this study reflects the results reported in previous studies carried out on physical education. This may be attributed to the traditional sports culture, assuming that the imposition of practices that predominantly include and embrace these teaching methods in coach and physical education teacher training programs originates from the traditional sports culture. Therefore, this may lead to the perpetuation of these existing teaching methods (57).

In the literature, community-based learning studies are recommended in order to enhance coaches' capacity to apply their knowledge and methods (55, 56). Collaborative learning environments designed based on this learning theory are known to be effective in providing quality learning environments for coaches [e.g., (58–60)].

In response to the second research question, while there was no significant gender-based difference in experienced coaches' usage levels and value perceptions of teaching methods (*p* > 0.05), experienced male and female physical education teachers indicated that they most frequently use the Exercise (B) and Learner-designed Individual Program (I) methods and they value these methods. However, they mentioned not using the Self-Check (D) and Inclusion (E) methods, although they value them. The common use of these methods by both male and female teachers is presumed to be influenced by the teaching environments or the number of students in the classes. The literature suggests similarities in teachers' individual assessments and general perceptions of teaching methods (10, 48). Considering the coaches, the focus is more on coach-athlete relationships, covering all dimensions. The important point here is that they were not examined considering the gender. Therefore, future studies should clarify the gender-based usage levels and value perceptions of teaching methods by coaches and physical education teachers.

Regarding the comparison of coaches' usage levels and value perceptions of teaching methods by their professional experience, significant differences were observed in the Exercise (B) method and its value perception between those with the experience of 10 years and those with the experience of 11 years or more, as well as in the Learner-designed Individual Program (I) and Learner-Initiated (J) methods between those with 5 years of experience and those with 10 years of experience. However, no significant difference was observed in other value perceptions. It is thought that coaches' specialization in a particular sports discipline and routine use of methods over time might lead to changes in their value perceptions. Coaches should have a good understanding of various teaching methods (3, 61) and, more importantly, theoretical knowledge to apply these methods appropriately in harmony with athletes' different learning needs (43, 62, 63). Evaluating the teaching methods coaches currently implement in their practices in a specific sports context is very important to improve their teaching capacities in line with these considerations.

Furthermore, considering physical education teachers, significant differences are observed in the “Command (A)” method between those with 10 years and 11 years and above of experience. For the “Self-Check (D),” “Guided discovery (F),” and “Problem-Solving: Single Solution (G)” methods, significant differences are noted between those with an experience of 5 years and those with an experience of 11 years or more. Regarding value perceptions, for “Exercise (B)” and “Problem-Solving: Single Solution (G),” significant differences were observed between those with an experience of 5 years and those with an experience of 11 years and above. For “Guided discovery (F),” “Problem-Solving: Creating Different Paths (H),” and “Learner-Initiated (J),” value perceptions exhibited significant differences across all years of experience. Moreover, it is evident that the most used method was found to be “Command (A),” whereas the most highly valued method was “Guided discovery (F).” The increase in experience is associated with changes in the methods used and value perceptions, highlighting the ergonomic utilization of knowledge and skills. In conclusion, in a changing and evolving world, applying the same teaching methods or techniques to athletes and students in each era repeatedly may not be appropriate.

## 5 Limitation

The limitations of the present study should be considered when interpreting the results achieved here. As scholars in Sports Sciences, we recommend that future studies examining the teaching provided by coaches and physical education teachers should incorporate systematic field observations and utilize qualitative inquiry to gain a more comprehensive understanding of the application capacities of these two groups' methods. Secondly, the data collection process was confined to three cities in the Thrace region of Turkey. Expanding the study to include a larger population from diverse coaching and physical education teaching contexts would add depth to the research.

## 6 Conclusion and recommendations

In conclusion, understanding the experiences and perceptions of coaches and physical education teachers regarding teaching methods is important for several reasons. First of all, gaining insight into teachers' experiences and perceptions of different styles is anticipated to lay a foundation that can be utilized in designing pre-service and in-service programs for teachers. These programs can be designed to encourage more effective use of commonly employed methods or to assist the adoption of new methods. Future research can be organized in a way to encourage the adoption and implementation of new teaching methods. Developing fundamental perceptions regarding the capacity of teaching methods to achieve different objectives can provide insights into educators' understanding of pedagogical knowledge. Informing sports education programs for coaches and physical education teachers about which teaching methods are most taught in their educational domains and how pre-service sports instructors can better prepare to surpass current practices can be facilitated by this understanding.

## Data Availability

The original contributions presented in the study are included in the article/Supplementary Material, further inquiries can be directed to the corresponding author.
